# Prognostic significance of NEK2 in human solid tumors: a systematic review and meta-analysis

**DOI:** 10.1042/BSR20180618

**Published:** 2019-01-18

**Authors:** Xichen Wang, Kang Chen, Haipeng Liu, Zeping Huang, Xiao Chen, Lanning Yin

**Affiliations:** 1 Department of General Surgery, Lanzhou University Second Hospital, Lanzhou 730030, Gansu Province, China; 2 Key Laboratory of Digestive System Tumors of Gansu Province, Lanzhou 730030, Gansu Province, China

**Keywords:** meta-analysis, NIMA-related kinase 2, prognosis, solid tumor

## Abstract

A consensus about the prognostic role of NIMA-related kinase 2 (NEK2) expression in various solid tumors has not been made yet. Thus, this meta-analysis aimed to systematically assess the prognostic role of NEK2 expression in patients with solid tumors. The eligible studies were identified through searching PubMed, Web of Science, and EMBASE. The hazard ratios (HRs) with their corresponding 95% confidence intervals (CIs) were used to evaluate the link between NEK2 overexpression and overall survival (OS) and disease-free survival/recurrence-free survival (DFS/RFS) of patients with solid tumors. A total of 17 studies with 4897 patients were included in this meta-analysis. Among these studies, all of them explored the association between NEK2 expression and OS of patients with solid tumors. Our pooled analysis indicated that NEK2 overexpression was significantly related to adverse OS (HR = 1.66; 95% CI: 1.38–2.00; *P* = 0.001). Additionally, there were six studies with 854 patients that investigated the association between NEK2 expression and DFS/RFS. Our pooled result indicated that there was a substantial relationship between NEK2 overexpression and poorer DFS/RFS (HR = 2.00; 95% CI: 1.61–2.48; *P* = 0.003). In conclusion, our meta-analysis indicated that NEK2 may be a useful predictor of prognosis and an effective therapeutic target in solid tumors. Nevertheless, more high-quality studies are warranted to further support our conclusions because of several limitations in our meta-analysis.

## Introduction

Human solid tumors have been the main root of global mortality for many years and remains a worldwide health problem [1]. Despite tremendous progress made in diagnosis and therapy during the past decades, the oncological survival of patients with solid tumor is still unfavorable, especially in advanced stage. In current, clinicopathologic parameters that mainly include pathological grade and clinical stage are the main factors used to predict the prognosis of cancer patients. However, these factors do not often work as a reliable predictors of early diagnosis and individual prognosis, which imposes restriction on the efficiency of therapies for solid tumors. Thus, it is of extremely vital significance to identify novel cancer biomarkers that display more accuracy in predicting tumor progression and clinical outcomes.

It has been clearly verified that genetic instability could contribute to tumorigenesis by activating oncogenes and/or inactivating tumor suppressor genes [2]. Chromosome instability (CIN), a phenotype featured with a high proportion of gain and/or loss of whole or large fragments of chromosomes during each cell division, plays a critical role in initiating genetic instability [3]. In recent years, CIN was widely reported to be closely associated with carcinogenesis, tumor progression, and resistance to chemotherapy and radiotherapy [4,5]. The abnormality in cell division is implicated in CIN in malignant tumors [6]. Consistently, many cell division-associated proteins are overexpressed in various cancers and contribute to the initiation of CIN in tumor cells [7,8]. For instance, there is evidence demonstrating that the overexpression of never in mitosis (NIMA) related kinase 2 (NEK2), a member of the NIMA-related serine/threonine kinase family and a key component of centrosome, could cause CIN in tumor cells [9].

More important, a large number of studies showed that NEK2 overexpression occurred in many human solid tumors, including hepatocellular carcinoma (HCC) [10–16], colorectal cancer (CRC) [17–19], pancreatic ductal cancer [20], lung cancer [21,22], prostate cancer [23], breast carcinoma [24], and glioma [25,26]. Moreover, these studies also suggested that NEK2 overexpression was significantly correlated with more unfavorable prognosis of patients with solid tumors. Due to lack of the thorough analysis on the reliability and degree of the prognostic significance of NEK2 overexpression in solid tumors, we performed the present meta-analysis to further assess the association between NEK2 overexpression and oncological survival and the potential of NEK2 as a potential therapeutic target for patients with solid tumors.

## Materials and methods

This meta-analysis was performed based on the PRISMA statement issued in 2009 [27].

### Literature search

The eligible studies were identified by searching PubMed, EMBASE, and Web of science from inception to April 20, 2018. The search terms consisted of ‘NEK2,’ ‘NIMA-related kinase 2,’ ‘cancer(s),’ ‘carcinoma(s),’ ‘tumor(s),’ ‘neoplasm(s),’ ‘malignant,’ and ‘malignancy or malignancies’.

### Selection criteria

The studies were included according to the following inclusion criteria: (1) The studies explored the prognostic value of NEK2 expression in terms of overall survival (OS), disease-free survival (DFS), and recurrence-free survival (RFS) of patients with solid tumor; (2) The studies were written in English.

The studies were excluded based on any of the following exclusion criteria: (1) reviews, letters, conference abstracts, case reports, and non-clinical studies; (2) the studies focused on exploring the prognostic value of NEK2 expression in patients with non-solid tumors; (3) the studies did not provide sufficient data about the prognostic value of NEK2 expression.

### Data extraction

Two authors extracted relevant data independently. When there were inconsistencies between the two investigators with respect to data extraction, the other co-authors intervened and raised solutions. The main characteristics of the eligible studies included: the name of first author, publication year, case source, tumor type, sample size, tumor clinical stage, methods of detecting NEK2 expression, and cut-off of high NEK2 expression. Additionally, we extracted the hazard ratios (HR) and corresponding confidence interval (CI), which were used for assessing the association of EK2 expression with survival of patients with solid tumor, including OS, DFS, and RFS. If HRs for survival data were not presented directly in an study, they would be calculated using established methods provided by Tierney et al. [28].

### Statistical analysis

In this meta-analysis all statistical processes were fulfilled using STATA, version 12.0 (Stata Corporation, College Station, TX, USA). The pooled HRs and corresponding 95% CIs were used to evaluate the link between NEK2 expression and survival of patients with solid tumors. Because the present study was a prognosis meta-analysis that included retrospective studies, there was unavoidable heterogeneity. To address the heterogeneity, we performed the meta-regression and stratified analyses to explore the potential source of heterogeneity, as well as synthesized data using a random-effects meta-analysis. The stratified analysis were conducted according to tumor type, region (Asian/Non-Asian), the detection methods of NEK2 expression (IHC/qPCR/microarray), and analysis type (univariate/multivariate) to find out the potential sources of heterogeneity of the pooled HRs for OS. Besides, sensitivity analyses were also performed through sequentially deleting single study to further investigate the potential sources of heterogeneity of the pooled HRs for OS and meanwhile assess whether our pooled HRs for OS and DFS/RFS were robust. We evaluated the publication bias using Begge’s funnel plot [29] and Egger’s test [30]. When Begge’s funnel plot was symmetrical and meanwhile *P* value of Egger’s test was >0.05, no significant publication bias was considered to exist in the present meta-analysis. If there was significant publication bias, the trim and fill method was applied to explore whether the publication bias significantly affect the dependability of the results of our meta-analysis [31]. A *P* value less than 0.05 indicated that there was statistical significance.

## Results

### The literature search

**Figure 1:**
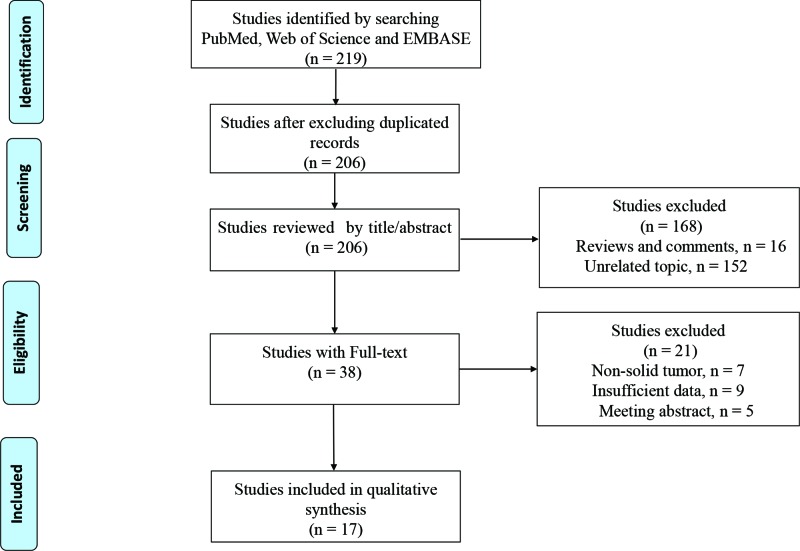
Flow diagram of study selection process

A total of 219 studies were identified through retrieving PubMed, EMBASE, and Web of science in the initial search. With 13 duplicated studies removed, 208 studies were left for title and abstract screening. A total of 168 studies were excluded owing to reviews and comments (*n* = 16) and the other topics (*n* = 152). As a result, 38 studies remained for full-text screening and in this process 21 studies were further excluded due to no data of interest (*n* = 9), meeting abstracts (*n* = 5), and non-solid tumors (*n* = 7). Finally, a total of 17 eligible studies were included in this meta-analysis [10–26]. The detail about the literature search and selection was displayed in Figure 1.

### The main characteristics of the eligible studies

A total of 13 studies were performed in China, 2 in Japan, 1 in UK [18], and 1 in Canada. Among the 17 eligible studies, 7 studies focused on HCC [10–16], 3 studies on CRC [17–19], 2 studies on lung cancer, 2 studies on glioma, 1 each study on breast cancer [24], pancreatic ductal adenocarcinoma (PDAC), [20] and prostate cancer [20]. A total of 12 included studies detected the expression level of NEK2 using IHC [10,11,13,15–18,20,22,23,25,26], 2 studies using qPCR [14,19], and 3 using microarray [12,21,24]. All the included studies with 4897 patients investigated the relationship between NEK2 expression and OS [10–26], and 6 studies with 854 patients explored the association between NEK2 expression and DFS/RFS [11,13,15,18,21,23]. More detailed information about the main characteristics was summarized in Table 1.

**Table 1:** The main characteristics of the included studies

First author/year/country	Tumor type	No. of patients	High NEK2 expression (*n*, %)	Sex (M/F)	TNM stage	Detection method of NEK2 expression	Cut-off of high NEK2 expression	Survival data	
Cappello, P./2013/Canada [24]	BC	2312	NR	NA	NA	Microarray	NA	OS	Univariate analysis
Fu, L./2017/China [10]	HCC	310	154 (49.68)	252/49	NA	IHC	More than 7 scores*	OS	Univariate analysis
Fu, S. J./2017/China [11]	HCC	100	69 (69.00)	NA	NA	IHC	More than 2 scores^†^	OS, DFS	Univariate analysis
Li, G./2017/China [12]	HCC	359	60 (16.71)	NA	NA	Microarray	NA	OS	Univariate analysis
Lin, S./2016/China [13]	HCC	104	60 (57.69)	32/12	I–III	IHC	More than 2 scores^†^	OS, RFS	Multivariate analysis
Wu, S.M./2016/China [14]	HCC	154	NR	76/78	I–III	qPCR	NA	OS	Univariate analysis
Wubetu, G. Y./2016/Japan [15]	HCC	50	25 (50.00)	34/16	I–IV	qPCR	More than the median value of mRNA expression	OS, RFS	Univariate analysis
Zhang, Y./2018/China [16]	HCC	259	98 (37.84)	NA	NA	IHC	More than 5 scores*	OS	Multivariate analysis
Lu, L./2015/China [17]	CRC	60	39 (65.00)	32/28	I–IV	IHC	More than 2 scores^‡^	OS	Univariate analysis
Neal, C. P./2014/UK [18]	CRC	103	89 (86.41)	57/46	I–IV	IHC	NA	OS, DFS,	Univariate analysis
Takahashi, Y./2013/Japan [19]	CRC	180	90 (50.00)	104/76	0–IV	qPCR	More than the median value of mRNA expression	OS	Multivariate analysis
Shi, Y.X./ 2016/China [21]	LC	349	175 (50.14)	159/190	NA	Microarray	NA	OS, RFS	Univariate analysis
Zhong, X./2014/China [22]	LC	270	70 (25.93)	192/78	I–IV	IHC	More than 240 scores^§^	OS	Multivariate analysis
Liu, H.J./2017/China [25]	GM	99	55 (55.56)	47/52	NA	IHC	More than 4 scores*	OS	Multivariate analysis
Wang, J./2017/China [26]	GM	44	25 (56.82)	NA	NA	IHC	NA	OS	Univariate analysis
Ning, Z./2014/China [20]	PDAC	136	74 (54.41)	72/64	I–IV	IHC	More than 4 scores*	OS	Univariate analysis
Zeng, Y. R./2015/China [23]	PC	148	74 (50.00)	NA	NA	IHC	NA	OS, RFS	Univariate analysis

**Abbreviations:** BC, breast cancer; CRC, colorectal cancer; DFS, disease-free survival; GM, glioma; HCC, hepatocellular carcinoma; HR, hazard ratio; LC, lung cancer; NR, not reported; PC, prostate cancer; PDAC, pancreatic duct adenocarcinoma; OS, overall survival; RFS, recurrence-free survival.

*The final score was assigned according to the result of multiplying the score of the staining intensity and the score of the proportion of stained malignant cells.

^†^The score was assigned according to the proportion of stained malignant cells.

^‡^The score was assigned according to the staining intensity of malignant tissues.

^§^The final score was assigned according to the result of multiplying the score of the staining intensity and the percentage of stained malignant cells.

### The combined analysis of the correlation between NEK2 expression and survival of patients with solid tumor

**Figure 2:**
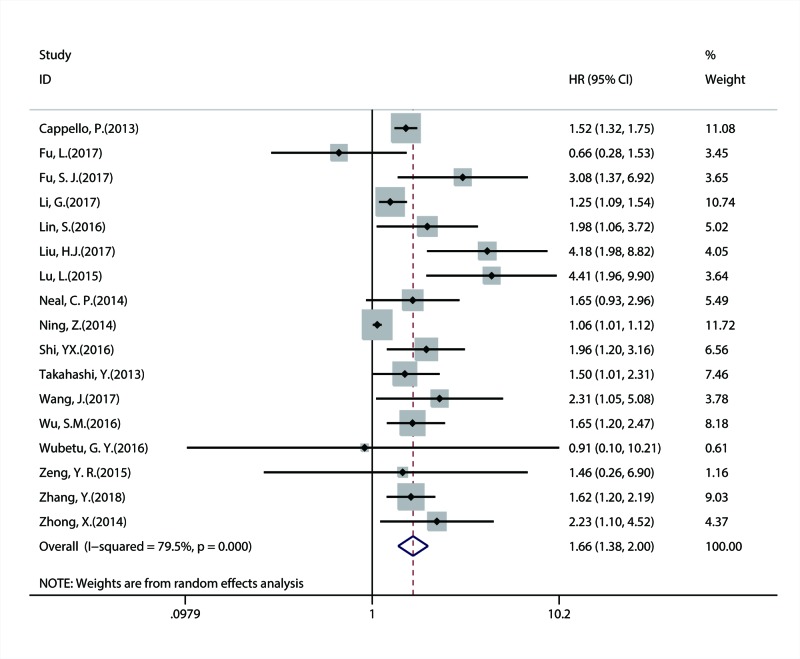
Forest plot of combined HR assessing the association between NEK2 expression and OS of patients with solid tumor

**Figure 3:**
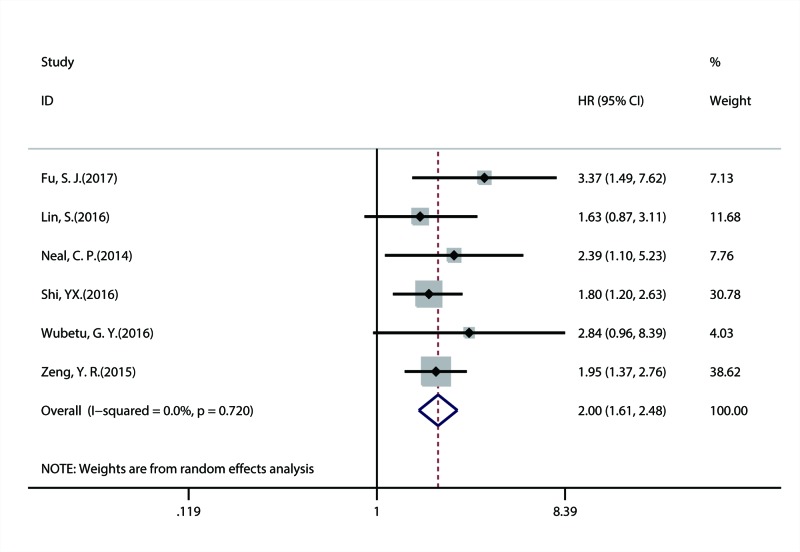
Forest plot of combined HR assessing the association between NEK2 expression and DFS/RFS of patients with solid tumor

A total of 17 studies with 4897 patients investigated the correlation between NEK2 overexpression and OS of patients with solid tumors. The combined result suggested that overexpression of NEK2 was significantly linked with more unfavorable OS (HR = 1.66; 95% CI: 1.38–2.00; *P* = 0.001) (Figure 2). In addition, six studies explored the association between NEK2 expression and DFS/RFS and included a total of 854 patients. Because DFS and RFS have similar statistical nature, they were merged together for the combined analysis. The combined result indicated that there was a substantial relationship between positive NEK2 expression and poorer DFS/RFS (HR = 2.00; 95% CI: 1.61–2.48; *P* = 0.003) (Figure 3).

### Subgroup and meta-regression analysis

To seek the potential sources of heterogeneity of the combined HR for OS, we performed the subgroup analysis based on tumor type, sample size, ethnicity, and detection methods of NEK2 expression and analysis type. In the subgroup analysis, we found that NEK2 overexpression was tightly related to worse OS of patients with HCC (HR = 1.50; 95% CI: 1.18–1.91; *P* < 0.01), CRC (HR = 2.03; 95% CI: 1.16–3.56; *P* = 0.03), glioma (HR = 3.15; 95% CI: 1.76–5.62; *P* < 0.01), lung cancer (HR = 2.04; 95% CI: 1.37–3.05; *P* < 0.01), breast cancer (HR = 1.52; 95% CI: 1.32–1.75; *P* < 0.01), and pancreatic duct adenocarcinoma (HR = 1.06; 95% CI: 1.01–1.12; *P* = 0.03) (Table 2). However, there was no significant association between NEK2 overexpression and worse OS of patients with prostate cancer (HR = 1.46; 95% CI: 0.28–7.52; *P* = 0.762) (Table 2). In addition, the subgroup analysis of ethnicity showed that NEK2 overexpression was significantly related to poorer OS of patients from both Asia (HR = 1.71; 95% CI: 1.337–2.12; *P* < 0.01) and non-Asia (HR = 1.53; 95% CI: 1.33–1.75; *P* < 0.01) (Table 2). The subgroup analysis by detection method showed that overexpression of NEK2 was significantly linked with worse OS regardless of methods (IHC: HR = 1.90, 95% CI = 1.35–2.68, *P* < 0.01; qPCR: HR = 1.57, 95% CI = 1.20–2.06, *P* < 0.01; microarray: HR = 1.45, 95% CI = 1.20–1.75, *P* < 0.01) (Table 2). The subgroup analysis by sample size showed that NEK2 overexpression was associated with worse OS in both small sample size group (<200) (HR = 1.95; 95% CI = 1.40–2.73; *P* < 0.01) and large sample size group (>200) (HR = 1.47; 95% CI = 1.23–1.76; *P* < 0.01) (Table 2). At last, the subgroup analysis by analysis type showed that NEK2 overexpression was closely associated with worse OS regardless of univariate analysis (HR = 1.54; 95% CI = 1.25–1.89; *P* < 0.01) or multivariate analysis [HR = 1.91; 95% CI = 1.43–2.56; *P* < 0.01 (Table 2)], which indicated NEK2 overexpression might be an independent risk factor for poor OS of patients with solid tumors. Overall, the results showed that the pooled HR for OS was stable and reliable, suggesting the five factors analyzed in subgroup analysis were not be the main source of heterogeneity. Additionally, we also performed meta-regression analysis to further determine whether the five factor could account for the majority of heterogeneity. The results showed that all the *P* values were more than 0.05 when we performed meta-regression using any one of the five factors as the covariate, which further confirmed that the five factors were not be main the source of heterogeneity.

**Table 2:** Subgroup and meta-regression analysis of the pooled HR for OS

	Subgroup analysis			Meta-regression
Factors	No. of studies	No. of patients	HR (95% CI)	*P* value
[1] Tumor type				0.596
Hepatocellular carcinoma	7	1336	1.50 (1.18, 1.91)	
Colorectal cancer	3	343	2.03 (1.16, 3.56)	
Glioma	2	143	3.15 (1.76, 5.62)	
Lung cancer	2	619	2.04 (1.37, 3.05)	
Breast cancer	1	2312	1.52 (1.32, 1.75)	
Pancreatic duct adenocarcinoma	1	136	1.06 (1.01, 1.12)	
Prostate cancer	1	148	1.46 (0.28, 7.52)	
[2] Ethnicity				0.767
Asian	15	2622	1.71 (1.37, 2.12)	
Non-Asian	2	2415	1.53 (1.33, 1.75)	
[3] Sample size				0.291
>200	6	3859	1.47 (1.23, 1.76)	
≤200	11	1178	1.95 (1.40, 2.73)	
[4] Detective methods				0.414
IHC	11	1633	1.90 (1.35, 2.68)	
qPCR	3	384	1.57 (1.20, 2.06)	
Microarray	3	3020	1.45 (1.20, 1.75)	
[4] Analysis type				0.296
Univariate	12	4125	1.54 (1.25, 1.89)	
Multivariate	5	912	1.91 (1.43, 2.56)	

### Sensitivity analysis

**Figure 4:**
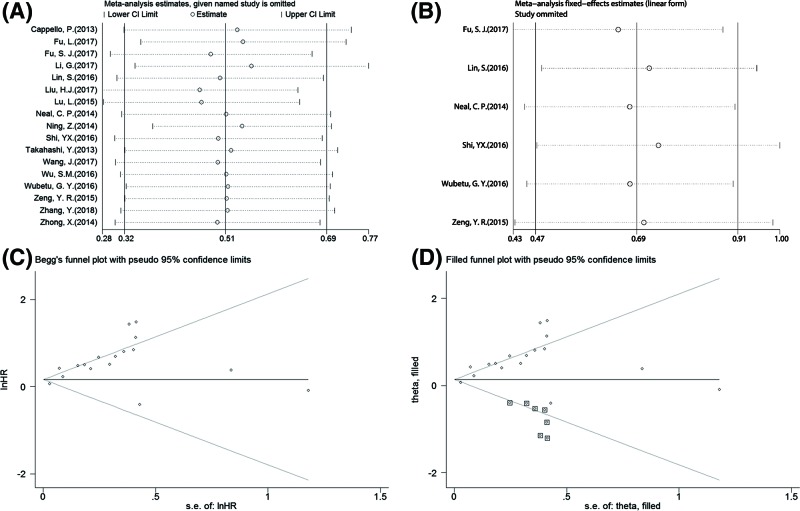
Sensitivity analysis and publication bias evaluation Sensitivity analysis of the combined HRs for OS (**A**) and DFS/RFS (**B**). Begg’s funnel plot of publication bias evaluation for the combined HR for OS (**C**). The adjusted Begg’s funnel plot of publication bias evaluation for the combined HR for OS from the trim-and-fill analysis (**D**).

Sensitivity analyses were performed to further explore the potential sources of heterogeneity, and meanwhile test the stability of the pooled HRs for OS and DFS/RFS. From the results, no substantial fluctuation of pooled HRs for OS (Figure 4A) and DFS/RFS (Figure 4B) was observed when omitting any individual study, which suggested that the pooled HRs for OS and DFS/RFS were robust.

### Publication bias

The Begg’s funnel plot and Egger’s tests were used to assess the potential publication bias of the included studies when combining the HR of OS. The Begg’s funnel plot showed significant asymmetry (Figure 4C), and it was verified by the result of Egger’s test (*P* < 0.001), which suggested that there was statistically significant publication bias of the included studies when combining the HR of OS. Hence, we performed the trim-and-fill analysis to determine whether the publication bias significantly affected the reliability of the combined HR for OS. The results showed that the reasonable number of the included studies should be 24 with 7 missing studies added into the pooled analysis, and meanwhile the updated funnel plot turned symmetric (Figure 4D). Furthermore, the adjusted pooled HR for OS also still suggested that NEK2 overexpression was significantly linked with worse OS, indicating that the potential publication bias did not significantly affected the reliability of our findings. Due to less than 10 eligible studies reporting about DFS/RFS, the publication bias was not assessed in this meta-analyses.

## Discussion

The present study is the first meta-analysis to systematically assess the relationship between NEK2 expression and survival of patients with solid tumor. Our combined results confirmed that there was a significant association between NEK2 overexpression and poor OS and DFS/RFS of patients with solid tumor, suggesting that NEK2 could be a useful prognostic predictor and a potential therapeutic target in patients with solid tumor.

Several oncogenesis mechanisms that may account for the link between NEK2 overexpression and unfavorable survival of patients with solid tumors have been reported. First of all, the roles of NEK2 overexpression in the tumor progression of HCC have been explored in a large number of recent studies. For instance, a study by Zhang et al. showed that NEK2 could promote the invasive ability of HCC cells by facilitating the epithelial–mesenchymal transition via several pathways including focal adhesion, VEGF, Hippo, and p53 pathways [32]. NEK2 overexpression could also enhance the proliferation, and inhibiting the apoptosis of HCC cells via the activation of MAPK pathway [33] and contribute to the migration, invasion, and angiogenesis of HCC cells by activating the AKT/NF-κB/MMP-2 pathway [12]. In addition to those signaling pathways above, several studies demonstrated that Wnt/β-catenin pathway plays a critical role in the NEK2 overexpression-meditated tumor progression. A study by Lin et al. suggested that NEK2 overexpression could contribute to the self-renewal property of HCC by Wnt/β-catenin pathway [13]. Besides, Lai et al. reported that NEK2 overexpression could promote cell cycle progression and proliferation of HCC cells and the activation of Wnt/β-catenin pathway was implicated in this process [34]. Moreover, a study by Wu et al. suggested that NKE2 overexpression could accelerate the tumor growth *in vivo*, and enhance the chemotherapeutic resistance properties of HCC cells by activating Wnt/β-catenin pathway [14].

Additionally, several studies have also investigated the roles of NEK2 overexpression in other solid tumors. For instance, Wang et al. reported that NEK2 overexpression could contribute to maintenance of glioma stem cells and induce radioresistance via stabilization of histone methyltransferase EZH2 [35]. On contrast, NEK2 knockdown could significantly inhibit the proliferation, colony formation, invasiveness, and *in vivo* growth of breast cancer cells [36]. Moreover, silencing NEK2 was demonstrated to sensitize triple-negative breast cancer cells to paclitaxel and doxorubicin [37], which indicated that NEK2 may be potential therapeutic target for breast cancer. Actually, recently many researchers have paid attention to the preclinical therapeutic effects of targeting NEK2 and reported that many NEK2 inhibitors could display *in vitro* and *in vivo* antitumor activities [38–40]. It has been widely recognized that glycolmetabolism plays crucial parts in cancer progression [41]. In particular, a recent study by Gu et al. indicated that NEK2 could promote aerobic glycolysis through modulating splicing of pyruvate kinase M (PKM) and then elevating the PKM2/PKM1 ratio in myeloma cells, which promotes myeloma cell proliferation [11]. To our best knowledge, the study by Gu et al. is the first one to investigate the effects of NEK2 on glycolmetabolism in myeloma. However, the effects of NEK2 on glycolmetabolism in solid tumors have not yet been explored so far. Thus, it may be interesting to explore the roles of NEK2 in regulating the glycolmetabolism in solid tumors. Taken together, these explorations of the role of NEK2 in solid tumors and the other malignancies implicated that NEK2 substantially affects the oncological survival of patients with solid tumors, which is consistent with our findings in the current meta-analysis.

There were several limitations in our meta-analysis, which should be considered when interpreting our findings. First, there was a certain heterogeneity in our meta-analysis and our subgroup and meta-regression analyses failed to identify the source of heterogeneity. The differences in some aspects maybe cause the heterogeneity, such as the cut-off values of NEK2 overexpression, follow-up time, and sexual ratio. Second, some HRs were calculated from the Kaplan–Meier curve, which might cause tiny statistical errors and then also introduce bias. Third, most of the included studies were performed in Asian, so it may not be reasonable to generate the findings from this meta-analysis to non-Asian population. Fourth, only studies published in English were included. Thus, potentially eligible studies published in other languages were not included, which maybe also introduce publication bias to some degree.

In conclusion, our meta-analysis indicated that NEK2 may be a useful predictor of prognosis and a potential therapeutic target in solid tumors. Nevertheless, more high-quality studies are warranted to further support our conclusions because of several limitations mentioned above.

## Abbreviations

CI: 95% confidence interval
CIN: chromosome instability
CRC: colorectal cancer
DFS: disease-free survival
HCC: hepatocellular carcinoma
HR: hazard ratio
NEK2: NIMA-related kinase 2
NIMA: never in mitosis
OS: overall survival
PDAC: pancreatic ductal adenocarcinoma
PKM: pyruvate kinase M

## References

[1] Siegel R.L., Miller K.D. and Jemal A. (2018) Cancer statistics, 2018. CA Cancer J. Clin. 68, 7–30 10.3322/caac.21442 29313949

[2] Lengauer C., Kinzler K.W. and Vogelstein B. (1998) Genetic instabilities in human cancers. Nature 396, 643–649 10.1038/25292 9872311

[3] Thompson S.L., Bakhoum S.F. and Compton D.A. (2010) Mechanisms of chromosomal instability. Curr. Biol. 20, R285–295 10.1016/j.cub.2010.01.034 20334839 PMC3781365

[4] Bakhoum S.F. and Compton D.A. (2012) Chromosomal instability and cancer: a complex relationship with therapeutic potential. J. Clin. Invest. 122, 1138–1143 10.1172/JCI59954 22466654 PMC3314464

[5] Tanaka K. and Hirota T. (2016) Chromosomal instability: a common feature and a therapeutic target of cancer. Biochim. Biophys. Acta 1866, 64–75 27345585 10.1016/j.bbcan.2016.06.002

[6] Bastians H. (2015) Causes of chromosomal instability. Recent Results Cancer Res. 200, 95–113 10.1007/978-3-319-20291-4_5 26376874

[7] de Carcer G. and Malumbres M. (2014) A centrosomal route for cancer genome instability. Nat. Cell Biol. 16, 504–506 10.1038/ncb2978 24875738

[8] Vitre B.D. and Cleveland D.W. (2012) Centrosomes, chromosome instability (CIN) and aneuploidy. Curr. Opin. Cell Biol. 24, 809–815 10.1016/j.ceb.2012.10.006 23127609 PMC3621708

[9] Zhou W., Yang Y., Xia J. (2013) NEK2 induces drug resistance mainly through activation of efflux drug pumps and is associated with poor prognosis in myeloma and other cancers. Cancer Cell 23, 48–62 10.1016/j.ccr.2012.12.001 23328480 PMC3954609

[10] Fu L., Liu S., Wang H. (2017) Low expression of NEK2 is associated with hepatocellular carcinoma progression and poor prognosis. Cancer Biomark. 20, 101–106 10.3233/CBM-170586 28759960

[11] Fu S.J., Chen J., Ji F. (2017) MiR-486-5p negatively regulates oncogenic NEK2 in hepatocellular carcinoma. Oncotarget 8, 52948–52959 28881785 10.18632/oncotarget.17635 PMC5581084

[12] Li G., Zhong Y., Shen Q. (2017) NEK2 serves as a prognostic biomarker for hepatocellular carcinoma. Int. J. Oncol. 50, 405–413 10.3892/ijo.2017.3837 28101574 PMC5238800

[13] Lin S., Zhou S., Jiang S. (2016) NEK2 regulates stem-like properties and predicts poor prognosis in hepatocellular carcinoma. Oncol. Rep. 36, 853 10.3892/or.2016.4896 27349376

[14] Wu S.M., Lin S.L., Lee K.Y. (2017) Hepatoma cell functions modulated by NEK2 are associated with liver cancer progression. Int. J. Cancer 140, 1581–1596 10.1002/ijc.30559 27925179

[15] Wubetu G.Y., Morine Y., Teraoku H. (2016) High NEK2 expression is a predictor of tumor recurrence in hepatocellular carcinoma patients after hepatectomy. Anticancer Res. 36, 757–762 26851035

[16] Zhang Y., Wang W., Wang Y. (2018) NEK2 promotes hepatocellular carcinoma migration and invasion through modulation of the epithelial-mesenchymal transition. Oncol. Rep. 39, 1023–1033 29399700 10.3892/or.2018.6224 PMC5802024

[17] Lu L., Zhai X. and Yuan R. (2014) Clinical significance and prognostic value of Nek2 protein expression in colon cancer. Int. J. Clin. Exp. Pathol. 8, 15467–15473 PMC4713702 26823916

[18] Neal C.P., Fry A.M., Moreman C. (2014) Overexpression of the Nek2 kinase in colorectal cancer correlates with beta-catenin relocalization and shortened cancer-specific survival. J. Surg. Oncol. 110, 828–838 10.1002/jso.23717 25043295

[19] Takahashi Y., Iwaya T., Sawada G. (2013) Up-regulation of NEK2 by MicroRNA – 128 methylation is associated with poor prognosis in colorectal cancer. Ann. Surg. Oncol. 21, 205–212 10.1245/s10434-013-3264-3 24046120

[20] Zhen N., Wang A., Liang J. (2014) Abnormal expression of Nek2 in pancreatic ductal adenocarcinoma: a novel marker for prognosis. Int. J. Clin. Exp. Pathol. 7, 2462 24966957 PMC4069945

[21] Shi Y.X., Yin J.Y., Shen Y., Zhang W., Zhou H.H. and Liu Z.Q. (2017) Genome-scale analysis identifies NEK2, DLGAP5 and ECT2 as promising diagnostic and prognostic biomarkers in human lung cancer. Sci. Rep. 7, 8072 10.1038/s41598-017-08615-5 28808310 PMC5556079

[22] Zhong X., Guan X., Liu W. and Zhang L. (2014) Aberrant expression of NEK2 and its clinical significance in non-small cell lung cancer. Oncol. Lett. 8, 1470 10.3892/ol.2014.2396 25202351 PMC4156209

[23] Zeng Y.R., Han Z.D., Wang C. (2015) Overexpression of NIMA-related kinase 2 is associated with progression and poor prognosis of prostate cancer. BMC Urol. 15, 90 10.1186/s12894-015-0085-7 26320076 PMC4553013

[24] Cappello P., Blaser H., Gorrini C. (2013) Role of Nek2 on centrosome duplication and aneuploidy in breast cancer cells. Oncogene 33, 2375–2384 10.1038/onc.2013.183 23708664

[25] Liu H., Liu B., Hou X. (2017) Overexpression of NIMA-related kinase 2 is associated with poor prognoses in malignant glioma. J. Neurooncol. 132, 1–9 10.1007/s11060-017-2401-4 28321704

[26] Wang J., Cheng P., Pavlyukov M.S. (2017) Targeting NEK2 attenuates glioblastoma growth and radioresistance by destabilizing histone methyltransferase EZH2. J. Clin. Invest. 127, 3075 10.1172/JCI89092 28737508 PMC5531394

[27] Moher D., Liberati A., Tetzlaff J., Altman D.G. and Group P. (2010) Preferred reporting items for systematic reviews and meta-analyses: the PRISMA statement. Int. J. Surg. 8, 336–341 10.1016/j.ijsu.2010.02.007 20171303

[28] Tierney J.F., Stewart L.A., Ghersi D., Burdett S. and Sydes M.R. (2007) Practical methods for incorporating summary time-to-event data into meta-analysis. Trials 8, 16 10.1186/1745-6215-8-16 17555582 PMC1920534

[29] Begg C.B. and Mazumdar M. (1994) Operating characteristics of a rank correlation test for publication bias. Biometrics 50, 1088–1101 7786990

[30] Egger M., Davey Smith G., Schneider M. and Minder C. (1997) Bias in meta-analysis detected by a simple, graphical test. BMJ 315, 629–634 9310563 10.1136/bmj.315.7109.629 PMC2127453

[31] Duval S. and Tweedie R. (2000) Trim and fill: a simple funnel-plot-based method of testing and adjusting for publication bias in meta-analysis. Biometrics 56, 455–463 10.1111/j.0006-341X.2000.00455.x 10877304

[32] Zhang Y., Wang W., Wang Y. (2018) NEK2 promotes hepatocellular carcinoma migration and invasion through modulation of the epithelial-mesenchymal transition. Oncol. Rep. 39, 1023–1033 29399700 10.3892/or.2018.6224 PMC5802024

[33] Zhang M.X., Xu X.M., Zhang P. (2016) Effect of silencing NEK2 on biological behaviors of HepG2 in human hepatoma cells and MAPK signal pathway. Tumor Biol. 37, 2023–2035 10.1007/s13277-015-3993-y 26337275

[34] Lai X.B., Nie Y.Q., Huang H.L. (2017) NIMA-related kinase 2 regulates hepatocellular carcinoma cell growth and proliferation. Oncol. Lett. 13, 1587–1594 10.3892/ol.2017.5618 28454295 PMC5403431

[35] Wang J., Cheng P., Pavlyukov M.S. (2017) Targeting NEK2 attenuates glioblastoma growth and radioresistance by destabilizing histone methyltransferase EZH2. J. Clin. Invest. 127, 3075–3089 10.1172/JCI89092 28737508 PMC5531394

[36] Tsunoda N., Kokuryo T., Oda K. (2009) Nek2 as a novel molecular target for the treatment of breast carcinoma. Cancer Sci. 100, 111–116 10.1111/j.1349-7006.2008.01007.x 19038001 PMC11158353

[37] Lee J. and Gollahon L. (2013) Nek2-targeted ASO or siRNA pretreatment enhances anticancer drug sensitivity in triplenegative breast cancer cells. Int. J. Oncol. 42, 839–847 23340795 10.3892/ijo.2013.1788 PMC3597451

[38] Fang Y., Kong Y., Xi J. (2016) Preclinical activity of MBM-5 in gastrointestinal cancer by inhibiting NEK2 kinase activity. Oncotarget 7, 79327–79341 10.18632/oncotarget.12687 27764815 PMC5346717

[39] Khanfar M.A., Banat F., Alabed S. and Alqtaishat S. (2017) Discovery of potent NEK2 inhibitors as potential anticancer agents using structure-based exploration of NEK2 pharmacophoric space coupled with QSAR analyses. Mol. Divers. 21, 187–200 10.1007/s11030-016-9696-5 27599492

[40] Xi J.B., Fang Y.F., Frett B. (2017) Structure-based design and synthesis of imidazo[1,2-a]pyridine derivatives as novel and potent Nek2 inhibitors with in vitro and in vivo antitumor activities. Eur. J. Med. Chem. 126, 1083–1106 10.1016/j.ejmech.2016.12.026 28039836

[41] Kato Y., Maeda T., Suzuki A. and Baba Y. (2018) Cancer metabolism: new insights into classic characteristics. Jpn. Dent. Sci. Rev. 54, 8–21 10.1016/j.jdsr.2017.08.003 29628997 PMC5884251

